# Phosphomimetic mutations near active sites of proteins in *Thermus thermophilus* suggest a widespread regulatory mechanism

**DOI:** 10.1002/2211-5463.70103

**Published:** 2025-09-15

**Authors:** Anzu Nishiwaki, Hiroki Okanishi, Yoshikatsu Kanai, Ryoji Masui

**Affiliations:** ^1^ Graduate School of Science Osaka Metropolitan University Japan; ^2^ Graduate School of Medicine Osaka University Japan; ^3^ Integrated Frontier Research for Medical Science Division Institute for Open and Transdisciplinary Research Initiative, Osaka University Japan; ^4^ Department of Metabolic Reprogramming and Signal Regulation Premium Research Institute for Human Metaverse Medicine (WPI‐PRIMe), Osaka University Japan

**Keywords:** nucleoside monophosphate kinase, phosphomimetic mutation, protein phosphorylation, *Thermus thermophilus*

## Abstract

In *Thermus thermophilus*, an aerobic Gram‐negative eubacterium used as a model organism, more than half of the phosphorylation sites identified by proteomic analysis are located near the ligand‐binding site, including the active site, of the enzyme in the three‐dimensional structure. We investigated the effect of these phosphorylation events on the activity of six enzymes (three nucleoside monophosphate kinases, isocitrate kinase, malate dehydrogenase and inorganic pyrophosphatase) by introducing phosphomimetic mutations, Glu, into the phosphorylation sites. All phosphomimetic mutants showed severely reduced activity compared with the wild‐type, particularly in the turnover number. The proteins analyzed in this study belong to different families and have various functions. This suggests that there is a widespread mechanism by which phosphorylation of amino acid residues near the active site reduces enzyme activity independent of the protein family and function.

## Graphical abstract

In *Thermus thermophilus*, more than half of the phosphorylation sites identified by proteomic analysis are located near the active site. All phosphomimetic mutants of phosphosites of six enzymes belonging to different families showed severely reduced activity compared with the wild‐type, particularly in the turnover number.

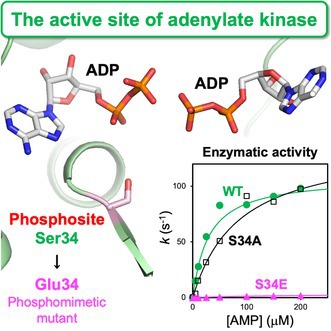

**Abbreviations**
NMPK: nucleoside monophosphate kinase
PMSF: phenylmethylsulfonyl fluoride
SASA: solvent accessible surface area
ttAMPK: *T. thermophilus* AMP kinase
ttCMPK: *T. thermophilus* CMP kinase
ttIDH: *T. thermophilus* isocitrate dehydrogenase
ttMDH: *T. thermophilus* malate dehydrogenase
ttPPase: *T. thermophilus* inorganic pyrophosphatase
ttUMPK: *T. thermophilus* UMP kinase
WT: wild‐type

Protein phosphorylation is a crucial mechanism involved in most cellular activities. With the development of phosphoproteomic techniques, protein phosphorylation has been studied extensively. However, it remains unknown whether most phosphosites play a role in a cellular process. From the protein chemistry point of view, understanding how phosphorylation affects the structure and function of a protein remains an important issue. In other words, one of the major challenges in protein phosphorylation research is to elucidate the functional relevance of each phosphosite [1]. Upon this challenge, structural information is indispensable to address the structure–function relationship of phosphorylated proteins.

It has become clear that phosphorylation of Ser, Thr, and Tyr, which was previously thought to be found only in eukaryotes, also occurs in bacteria [2, 3, 4, 5, 6]. We have conducted phosphoproteome analysis of *Thermus thermophilus* HB8, an aerobic Gram‐negative eubacterium that grows at temperatures ranging from 50 to 82 °C [7]. This extreme thermophile is a model organism for the structural proteomics project [8], and its proteins are highly stable and suitable for structure–function studies. We identified 52 phosphopeptides isolated from 48 proteins and determined 46 distinct phosphosites [9]. In addition, mapping of the phosphorylation sites on the three‐dimensional structure revealed that more than half of the phosphorylation sites were located near the ligand‐binding site [9, 10]. These results lead to the hypothesis that phosphorylation of the ‘nearby’ site is involved in negative regulation of the enzymatic activity by inhibiting ligand binding.

Among the proteins with the identified phosphorylation sites in proximity to bound ligands are isocitrate dehydrogenase (ORF ID, TTHA1535), malate dehydrogenase (TTHA0536) and inorganic pyrophosphatase (TTHA1965), as well as three nucleoside monophosphate kinases (NMPKs), namely AMP kinase (TTHA1671), CMP kinase (TTHA0458) and UMP kinase (TTHA0859). NMPKs synthesize nucleotide diphosphates from nucleoside monophosphates using ATP [11]. AMP kinase and CMP kinase are conserved in most organisms, and amino acid sequence homology exists between eukaryotic and bacterial homologs [12]. Many structures of complexes with substrates, products, and their analogs have been determined [13, 14, 15]. Based on this structural information, substrate recognition and catalytic mechanisms have also been proposed [11]. On the contrary, prokaryotic UMP kinase belongs to a different amino acid kinase protein family from eukaryotic UMP kinase. Prokaryotic UMP kinase forms a hexamer and shows no similarity in sequence or 3D structure to AMP kinase or CMP kinase, which act as monomers. Many crystal structures of bacterial UMP kinase, including complexes with ligands, have been determined [16, 17].

The crystal structures of NMPKs from *T. thermophilus* have already been determined mainly through structural proteomics studies. For *T. thermophilus* CMPK (ttCMPK), the crystal structures in various liganded states have been determined [18]. For *T. thermophilus* AMPK (ttAMPK), only the apo form structure has been determined (PDB ID, 3CM0). For *T. thermophilus* UMPK (ttUMPK), the structures of the UMP–ADP complex have only recently been determined (8YH1). The structures of isocitrate dehydrogenase (ttIDH) and inorganic pyrophosphatase (ttPPase) from *T. thermophilus* have also been determined (2D1C and 2PRD, respectively), although that of malate dehydrogenase (ttMDH) has not. When the identified phosphorylation sites were mapped onto these three‐dimensional structures, it was found that all sites were located near the active site (substrate‐binding site) of each enzyme [9, 10] (Fig. 1). The substrates of these enzymes are phosphate group‐containing compounds. If a residue near the active site is phosphorylated, it is highly likely that the binding of a substrate with the same negative charge and the catalytic reaction with a phosphate group transferred will be affected and that the activity will be suppressed or inhibited.

**Fig. 1:**
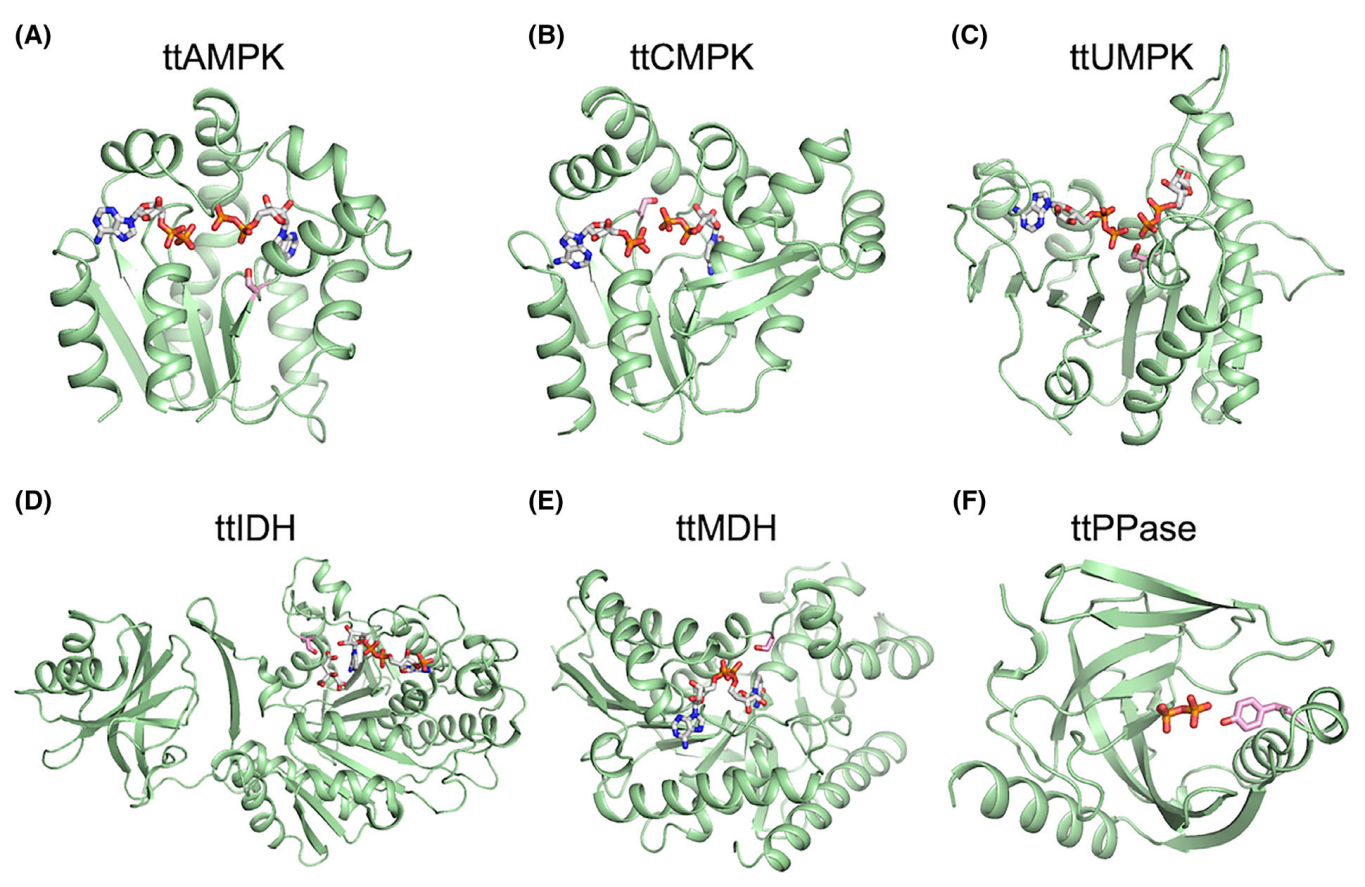
Structural mapping of the phosphosites identified in *T. thermophilus* enzymes. (A) A model structure of ttAMPK predicted using *E. coli* AMP kinase structure in complex with two ADP molecules (PDB ID, 7APU). (B) A crystal structure of ttCMPK‐CDP‐ADP complex (3AKC). (C) A crystal structure of ttUMPK‐UDP‐ADP complex (8YH1). (D) A crystal structure of ttIDH‐NADP^+^ complex (2D1C). (E) A model structure of ttMDH predicted using *Thermus flavus* malate dehydrogenase structure (1BMD) in complex with NAD^+^ molecule. (F) A crystal structure of ttPPase (2PRD). A pyrophosphate molecule in *E. coli* inorganic pyrophosphatase structure (2AU9) is also shown after structural alignment. Ligands are shown as sticks colored according to atom type. The side chains of the respective phosphosites are shown as sticks colored pink. Structure diagrams were drawn using the program PyMOL.

In this study, we examined whether phosphorylation near the active site affects enzymatic activity. All phosphomimetic mutants showed reduced activity compared with the wild‐type (WT), particularly the *k*_cat_ value.

## Materials and methods

### Preparation of the recombinant proteins

The expression plasmid for ttAMPK as well as the other five enzymes was obtained from the RIKEN Bioresource Research Center (Tsukuba, Japan). The expression plasmid of ttAMPK (pET‐3a/ttha1671) was introduced into *Escherichia coli* Rosetta2(DE3) (Novagen, Madison, WI, USA). The transformant was cultured at 37 °C for 24 h in an overexpression medium as previously described [19]. After centrifugation, the harvested cells were ultrasonicated on ice in buffer I (50 mm Tris–HCl, pH 7.5 and 150 mm NaCl) with 1 mm phenylmethylsulfonyl fluoride (PMSF). After heat treatment at 80 °C for 10 min, the supernatant was recovered by centrifugation (7600 ***g***) for 1 h at 4 °C. Ammonium sulfate was added to the supernatant up to 1.4 m, and the solution was loaded onto 13 mL of Toyopearl Butyl‐650M resin (Tosoh, Tokyo, Japan) pre‐equilibrated with buffer I containing 1.4 m ammonium sulfate. The resin was washed using the same buffer and eluted with a 160 mL gradient of 1.4–0 m ammonium sulfate in buffer I. The ttAMPK in the fraction was precipitated using 2.0 m ammonium sulfate. After centrifugation (7600 ***g***) for 1 h at 4 °C, the precipitant was dissolved in buffer I. The solution was loaded onto a HiLoad 16/600 Superdex 75 pg column (GE Healthcare Biosciences, Piscataway, NJ, USA) on an ÄKTA explorer system (GE Healthcare Biosciences). The fractions containing the target protein were concentrated using a Vivaspin concentrator (Sartorius AG, Göttingen, Germany) and stored at 4 °C. The protein concentration was determined on the basis of the absorbance at 280 nm using the molar extinction coefficient (5880 m ^−1^·cm^−1^) calculated through the previously described procedure [20].

The expression plasmid of ttCMPK (pET‐11a/ttha0458) was introduced into *E. coli* C41(DE3)pRARE2 [21]. The ttCMPK‐overexpressed cells suspended were heat‐treated at 70 °C and lysed in the same manner as ttAMPK. After centrifugation, the precipitant was dissolved in 50 mm Tris–HCl, pH 9.0. The solution was loaded onto 8 mL of Toyopearl SuperQ‐650M resin (Tosoh, Tokyo, Japan) pre‐equilibrated with 50 mm Tris–HCl, pH 9.0. The resin was washed using the same buffer and eluted with a 160 mL gradient of 0–1.0 m NaCl in 50 mm Tris–HCl, pH 9.0. The ttCMPK in the fraction was precipitated using 1.5 m ammonium sulfate. After centrifugation, the precipitant was dissolved in buffer I. The protein concentration was determined similarly using the molar extinction coefficient (23 205 m ^−1^·cm^−1^).

The *E. coli* C41(DE3)pRARE2 was transformed with the expression plasmid of ttUMPK (pET‐11a/ttha0859). The ttUMPK‐overexpressed cells suspended were heat‐treated at 70 °C and purified in essentially the same manner as ttAMPK. The protein concentration was determined using the molar extinction coefficient (14 595 m ^−1^·cm^−1^).

The transformant cells of *E. coli* C41(DE3)pRARE2 with the expression plasmid of ttIDH (pET‐11a/ttha1535) were subject to essentially the same procedure as for ttAMPK but without size exclusion chromatography. The protein concentration was determined using the molar extinction coefficient (33 600 m ^−1^·cm^−1^).

The expression plasmid of ttPPase (pET‐11a/ttha1965) was introduced into *E. coli* C41(DE3)pRARE2. The ttPPase‐overexpressed cells were subjected to essentially the same purification procedures as for ttAMPK except for the use of Toyopearl Phenyl‐650M resin instead of Toyopearl Butyl. The protein concentration was determined using the molar extinction coefficient (21 840 m ^−1^·cm^−1^).

The transformant cells of *E. coli* C41(DE3)pRARE2 with the expression plasmid of ttMDH (pET‐11a/ ttha0536) were cultured, ultrasonicated, and heat‐treated in a similar way to ttCMPK, and ttMDH was purified by Toyopearl Butyl‐650 M column. The protein concentration was determined using the molar extinction coefficient (33 390 m ^−1^·cm^−1^).

### Preparation of mutant proteins

The expression plasmids for respective mutants were constructed by PrimeSTAR Mutagenesis (Takara Bio, Kusatsu, Japan), using respective expression plasmids for the WT as a template and primers (Table 1). All mutant proteins were prepared using the same method as for the respective WT proteins.

**Table 1:** Primes for construction of mutated proteins in this study.

Primer	Sequence
ttAMPK_S34A_f	aagctcgccacgggggacatcctc
ttAMPK_S34A_r	ccccgtggcgagcttcttgaagccgag
ttAMPK_S34E_f	aagctcgaaacgggggacatcctccgg
ttAMPK_S34E_r	ccccgtttcgagcttcttgaagccgag
ttCMPK_S11A_f	gggcctgcggcctccggcaagag
ttCMPK_S11A_r	ggaggccgcaggcccgtctatggtcac
ttCMPK_S11E_f	gggcctgaagcctccggcaagagc
ttCMPK_S11E_r	ggaggcttcaggcccgtctatggtcac
ttUMPK_S11A_f	aagcttgcgggcgagttcctgacccg
ttUMPK_S11A_r	ctcgcccgcaagcttcaggagaacccg
ttUMPK_S11E_f	aagcttgaaggcgagttcctgacccg
ttUMPK_S11E_r	ctcgccttcaagcttcaggagaacccg
ttIDH_S98A_f	gagaaggcggccaacgtcaccctaag
ttIDH_S98A_r	gttggccgccttctccccgtagccc
ttIDH_S98E_f	gagaaggaagccaacgtcaccctaagg
ttIDH_S98E_r	gttggcttccttctccccgtagcccac
ttMDH_S236A_f	ggggccgcgagcgccgccagc
ttMDH_S236A_r	ggcggcgctcgcggccccccg
ttMDH_S236E_f	ggggccgaaagcgccgccagcgcc
ttMDH_S236E_r	ggcggcgctttcggccccccgggc
ttPPase_Y140F_f	gagacctttaaggcactcgaagccaag
ttPPase_Y140F_r	tgccttaaaggtctcaaagaagtgctg
ttPPase_Y140E_f	gagaccgagaaggcactcgaagccaag
ttPPase_Y140E_r	tgccttctcggtctcaaagaagtgctg

### Enzyme assay

The activity of ttAMPK, ttCMPK, and ttUMPK was measured at 25 °C by an enzyme‐coupled spectrophotometric assay [22]. The change in absorbance at 340 nm was measured with a Hitachi spectrophotometer, model U‐3000. AMP (0–200 μm) was reacted with 10 nm ttAMPK in 50 mm Tris–HCl (pH 7.5), 100 mm KCl, 1 mm ATP, 5 mm MgCl_2_, 0.3 mm phosphoenolpyruvate, 0.15 mm NADH, 20 units·mL^−1^ pyruvate kinase (Sigma‐Aldrich) and 10 units·mL^−1^ lactate dehydrogenase (TOYOBO). The kinetic parameters were determined using the Michaelis–Menten equation. For the ttCMPK assay, ttCMPK (40 nm) was reacted with CMP (0–200 μm) and 1 mm ATP. To determine *K*_m_ for ATP, the enzyme was reacted with 1 mm CMP and ATP (0–200 μm). For the ttUMPK assay, ttUMPK (100 nm) was reacted with UMP (0–200 μm) and 1 mm ATP. To determine *K*_m_ for ATP, the enzyme was reacted with 1 mm CMP and ATP (0–800 μm).

The activity of ttIDH was measured at 60 °C in 50 mm Tris‐HCl (pH 7.5), 100 mm KCl, 5 mm MgCl_2_, 10 nm ttIDH, 4 mm NADP^+^, and isocitrate (0–600 μm). The change in absorbance at 340 nm was measured with a spectrophotometer. To determine *K*_m_ for NADP^+^, the enzyme was reacted with 2 mm isocitrate and NADP^+^ (0–200 μm).

The activity of ttMDH was measured at 30 °C in 50 mm Tris–HCl (pH 7.5), 100 mm KCl, 5 mm MgCl_2_, 1 nm ttMDH, 0.15 mm NADH, and oxaloacetate (0–40 μm). The change in absorbance at 340 nm was measured with a spectrophotometer. To determine *K*_m_ for NADH, the enzyme was reacted with 25 μm oxaloacetate and NADH (0–100 μm).

The reaction of ttPPase was performed in 50 mm Tris–HCl (pH 7.5), 100 mm KCl, 2 mm MgCl_2_ with 3 nm ttPPase and sodium pyrophosphate (0–1.5 mm) at 60 °C for 5 min in 100 μL. A 70 μL aliquot was mixed with 140 μL of BIOMOL Green (Enzo Life Science, Plymouth, PA, USA) and incubated at 30 °C for 15 min. The release of free inorganic phosphate was determined by measuring the absorbance at 650 nm with a spectrophotometer.

### Sequence and structural data analysis

Sequence logos were generated with Weblogo3 [23] for the sequences from the Pfam seed datasets: PF00406 (AMP kinase), PF02224 (CMP kinase), PF00180 (isocitrate dehydrogenase), PF02866 (malate dehydrogenase) and PF18823 (inorganic pyrophosphatase) [24]. The sequence log for UMP kinase was generated for the 50 most diverse members of AAK_UMPK‐PyrH‐Ec in the Conserved Domain Database [25].

A model structure of ttAMPK was predicted by SWISS‐MODEL [26] using *E. coli* AMP kinase structure in complex with two ADP molecules (PDB ID, 7APU) as the template. Two ADP molecules in the *E. coli* enzyme are also shown after structural alignment. A model structure of ttMDH was predicted using *Thermus flavus* malate dehydrogenase structure (1BMD). NAD^+^ molecule in the *T. flavus* enzyme is also shown. For the ttPPase structure, a pyrophosphate molecule in *E. coli* inorganic pyrophosphatase structure (2 AU9) is also shown after structural alignment. The relative per‐residue solvent accessible surface area (SASA) of the mutated site was estimated by PyMOL (https://pymol.org/2/) using these structures. The free energy difference between the WT and mutant protein (ΔΔG) was calculated by STRUM [27]. Model structures of the phosphomimetic mutant and wild‐type proteins were predicted by AlphaFold server (https://alphafoldserver.com/). Structure diagrams were drawn using the program PyMOL.

## Results

**Fig. 2:**
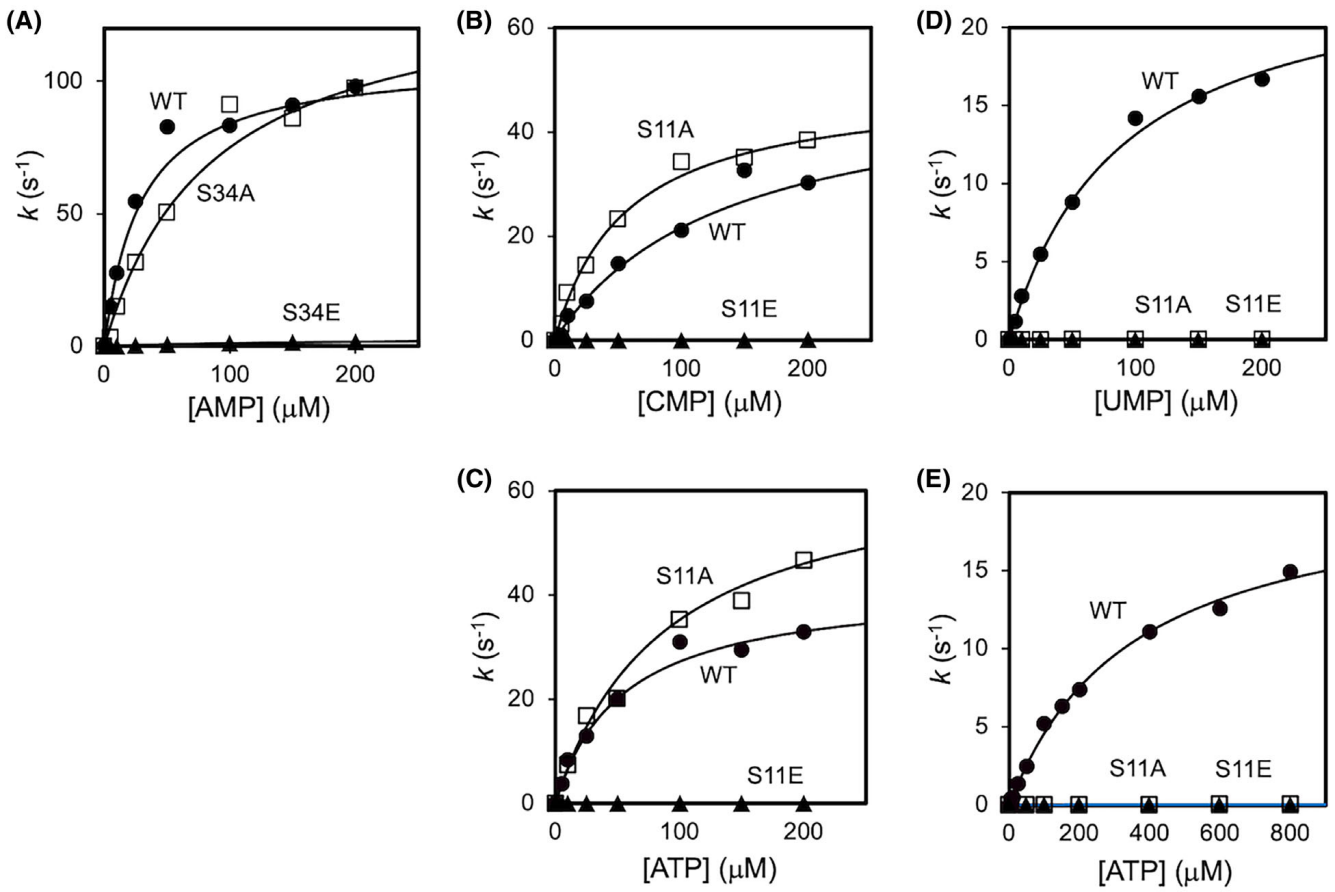
Enzymatic activities of NDPKs. (A) ttAMPK. (B, C) ttCMPK. (D, E) ttUMPK. The ordinates represent the reaction rate constants (*k*). Symbols: WT, circles; the mutant that contained Ala substituted for the Ser phosphorylation site, squares; and the mutant that contained Glu substituted for the Ser phosphorylation site, triangles. The theoretical curves were calculated using the kinetic parameters given in Table 2.

**Fig. 3:**
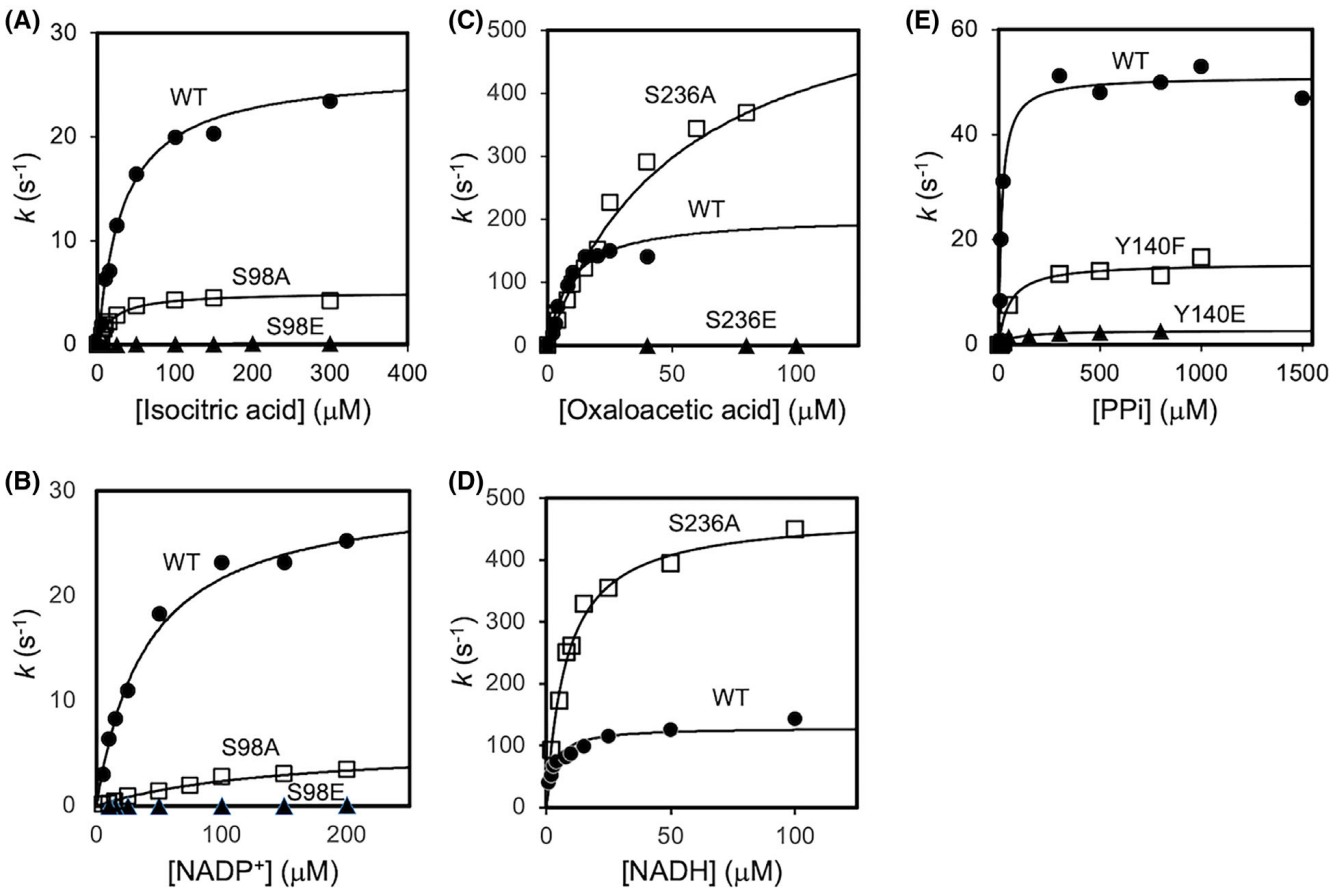
Enzymatic activities of ttIDH (A, B), ttMDH (C, D) and ttPPase (E). Symbols: WT, circles; the mutant that contained Ala (or Phe) substituted for the Ser phosphorylation site, squares; and the mutant that contained Glu substituted for the Ser phosphorylation site, triangles. The theoretical curves were calculated using the parameters given in Table 2.

We focused on the proteins containing the phosphorylation sites near the active site previously identified by our phosphoproteome analysis [9]. The selected target proteins were three NMPKs, ttIDH, ttMDH, and ttPPase (Fig. 1). We prepared mutants of these six enzymes by introducing phosphomimetic mutations (Glu) and Ala or Phe into the phosphorylation sites, and we compared their activity with that of the WT to infer the effects of phosphorylation (Figs 2 and 3, Table 2). Substitution by Glu will introduce a negative charge and potential steric hindrance. Therefore, we generated the Ser‐to‐Ala or Tyr‐to‐Phe mutants to estimate the effects of introducing a negative charge, in other words, to examine the importance of the original Ser or Tyr residue in respective proteins.

**Table 2:** Kinetic constants for ttNMPKs, ttIDH, ttMDH, and ttPPase. N.D., not determined. Values are shown as mean ± SD. Two *k* ^cat^ values were obtained from measurements under conditions that differed in excess substrate in the case of a bisubstrate enzyme. Asterisks indicate values significantly different from those of respective WT enzymes: *, *P* < 0.05; **, *P* < 0.01, and ***, *P* < 0.001.

	*K*_m_ (μM)		*k*_cat_ (s^−1^)		
ttAMPK	*K*_m_^AMP^				
WT	23 ± 1		111 ± 3		
S34A	71 ± 2*		142 ± 4		
S34E	153 ± 1***		3.7 ± 0***		
ttCMPK	*K*_m_^CMP^	*K*_m_^ATP^			
WT	61 ± 19	53 ± 1	54 ± 16	/	40 ± 1
S11A	53 ± 3	73 ± 16	57 ± 8	/	59 ± 4
S11E	79 ± 38	60 ± 14	0.11 ± 0.04*	/	0.05 ± 0.01***
ttUMPK	*K*_m_^UMP^	*K*_m_^ATP^			
WT	74 ± 10	214 ± 7	21 ± 1	/	14 ± 0
S11A	66 ± 1	732 ± 206*	0.07 ± 0.01***	/	0.04 ± 0.02***
S11E	N.D.	N.D.	N.D.	/	N.D.
ttIDH	*K*_m_^isocitrate^	*K*_m_^NADP+^			
WT	34 ± 2	39 ± 1	26 ± 1	/	30 ± 2
S98A	14 ± 6	150 ± 2***	4.4 ± 0.1***	/	5.0 ± 0.9**
S98E	178 ± 28*	144 ± 21*	0.19 ± 0.02***	/	0.12 ± 0**
ttMDH	*K*_m_^oxaloacetate^	*K*_m_^NADH^			
WT	8.6 ± 0.4	2.7 ± 0.9	203 ± 5	/	126 ± 14
S236A	52 ± 2***	7.6 ± 0.2*	622 ± 8*	/	468 ± 15**
S236E	19800 ± 100***	N.D.	12 ± 1***	/	N.D.
ttPPase	*K*_m_^PPi^				
WT	17 ± 2		62 ± 11		
Y140F	53 ± 0***		19 ± 3		
Y140E	62 ± 25		3.1 ± 0.4*		

The activity of ttAMPK, one of the NMPKs, was measured by changing AMP concentration under the condition of ATP excess. The initial velocity of the reaction in which ADP was generated was calculated from the absorbance change obtained in an enzyme‐coupled assay and plotted against the substrate concentration. Then, *K*_m_ and *k*_cat_ values of the WT and mutants were obtained using the Michaelis–Menten equation. The activity was significantly reduced in the phosphomimetic mutant S34E (Fig. 2A). The *K*_m_ and *k*_cat_ values of WT were 23 μm and 111 s^−1^, respectively. The *K*_m_ of S34A was 71 μm, which was about three times higher, and the *k*_cat_ was 142 s^−1^, which was about 1.3 times higher than WT. This indicates that S34A binds to the substrate less easily than WT, but the catalytic activity is almost unchanged. On the other hand, the *K*_m_ of S34E was 153 μm, which was about 6.6 times higher than WT, and the *k*_cat_ was 3.7 s^−1^, which was approximately 3%. It was found that S34E binds to the substrate even less easily than S34A, and catalysis is also much slower than WT.

Next, we analyzed ttCMPK similarly (Fig. 2B,C). By varying CMP concentration under ATP excess conditions, the *K*_m_(CMP) and *k*_cat_ values of WT were determined to be 61 μm and 54 s^−1^, respectively, whereas the *K*_m_(CMP) and *k*_cat_ of S11A were 53 μm and 57 s^−1^, respectively. This indicates that the substitution with Ala had little effect on either *K*_m_(CMP) or *k*_cat_. In contrast, the *K*_m_(CMP) of S11E was 79 μm and *k*_cat_ was 0.11 s^−1^, indicating that the binding affinity of the phosphomimetic mutant to the substrate CMP was unchanged compared with WT, but the turnover number was slowed down approximately 490 times. Furthermore, under conditions of excess CMP, the *K*_m_(ATP) and *k*_cat_ of WT were 53 μm and 40 s^−1^, whereas those of S11A were 73 μm and 59 s^−1^, and those of S11E were 60 μm and 0.05 s^−1^. These results show little change in the binding affinity for ATP.

ttUMPK was also analyzed in the same manner as the above two enzymes (Fig. 2D,E). Under ATP excess conditions, *K*_m_(UMP) and *k*_cat_ of WT were 74 μm and 21 s^−1^, whereas *K*_m_(UMP) and *k*_cat_ of S11A were 14 μm and 0.07 s^−1^, respectively. In the case of ttUMPK, a significant decrease in *k*_cat_ was observed even in the Ala‐substituted mutant, indicating the importance of the Ser residue at this position in catalysis. In addition, the activity of S11E was very weak, and the parameters could not be calculated based on the Michaelis–Menten equation. Under UMP excess conditions, *K*_m_(ATP) and *k*_cat_ of WT were 214 μm and 14 s^−1^. In comparison, *K*_m_(ATP) and *k*_cat_ of S11A were 732 μm and 0.04 s^−1^. Thus, although S11A has almost the same binding affinity for both substrates as WT, the catalysis is approximately 320‐fold slower, indicating the functional importance of Ser11.

In summary, the activities of the phosphomimetic mutants of the three NMPKs examined were significantly reduced. This is thought to be due to the inhibition of the phosphotransfer reaction from ATP.

We also prepared phosphomimetic mutants of ttIDH, ttMDH, and ttPPase and compared their activities with those of the WT (Fig. 3, Table 2). Under conditions of excess NADP^+^, the *K*_m_(isocitrate) and *k*_cat_ of the WT ttIDH were 34 μm and 26 s^−1^, respectively, whereas the *K*_m_(isocitrate) and *k*_cat_ of S98A were 14 μm and 4.4 s^−1^, respectively, showing a slight decrease in *k*_cat_ (Fig. 3A,B). In addition, the *K*_m_(isocitrate) and *k*_cat_ of S98E were 178 μm and 0.19 s^−1^, respectively, showing a 5‐fold decrease in affinity for isocitrate compared with WT, but the decarboxylation reaction was approximately 130‐fold slower. Under conditions of excess isocitrate, the *K*_m_(NADP^+^) and *k*_cat_ of WT were 39 μm and 30 s^−1^, respectively. In contrast, the *K*_m_(NADP^+^) and *k*_cat_ of S98A were 150 μm and 5.0 s^−1^, respectively, and the *K*_m_(NADP^+^) and *k*_cat_ of S98E were 144 μm and 0.12 s^−1^, respectively, and the affinity for NADP^+^ was comparable to that of S98A.

The binding affinity of ttMDH with the substrate was also significantly affected by the phosphomimetic mutation (Fig. 3C,D). The *K*_m_(oxalate) and *k*_cat_ of S236A were 52 μm and 622 s^−1^, respectively, compared with 8.6 μm and 203 s^−1^ for WT, and the *K*_m_ (oxalate) and *k*_cat_ of S236E were 1.98 × 10^4^ μm and 12 s^−1^ for S236E. S236E had a substrate affinity approximately 2300‐fold lower than that of WT, and catalytic activity was approximately 17‐fold slower. Furthermore, the *K*_m_(NADH) and *k*_cat_ of S236A were 7.6 μm and 468 s^−1^, respectively, compared with 2.7 μm and 126 s^−1^ for WT. As for S236E, the activity was very small and could not be measured with high accuracy.

We prepared the phosphomimetic mutant of ttPPase and the mutant in which the phosphorylation site Tyr140 was replaced by Phe and measured their activity for pyrophosphate (PPi) (Fig. 3E). The *K*_m_ and *k*_cat_ of WT were 17 μm and 62 s^−1^, respectively, whereas the *K*_m_ and *k*_cat_ of Y140F were 53 μm and 19 s^−1^, respectively. The *K*_m_ and *k*_cat_ of Y140E were 62 μm and 3.1 s^−1^, respectively. This indicates that the catalytic activity of Y140E is approximately 20 times slower than that of WT.

To assess the effects of phosphomimetic mutations on the activity, we structurally characterized the phosphorylation site, Ser or Tyr, of respective proteins. Figure 5 shows the possible interactions of the phosphosite with ligands and other residues (left rows) and the local electrostatic potential maps around the site (middle rows). In addition, we predicted a model structure of the phosphomimetic mutant protein by AlphaFold3 [28] and superimposed it on the WT structure (Fig. 5, right rows). It should be noted that a model structure of the WT enzyme was also predicted for comparison with the mutant structure. To examine the effect of the mutation, we predicted a protein‐ligand complex structure except for ttPPase. It should be noted that two ADP molecules were utilized as a ligand for ttCMPK and ttUMPK instead of CDP and UDP, respectively, because only a limited variety of ligands are available for the AlphaFold server. The pLDDT and PAE scores indicated the models are confident predictions (Fig. S1).

Ser34 of ttAMPK makes interactions with the phosphate moiety of two ADP molecules and side chains of Asp37 and Asp83 (Fig. 5A). The location of the ligands and those side chains is unaltered, except for Asp37, even in the S34E structure. Ser11 of ttCMPK interacts with ADP/ATP and the side chains of As171 and Asp172 (Fig. 5B). Substitution of this residue for Glu is not expected to alter the structure and its global stability. Also for ttMDH and ttPPase, the structures and their stability are not predicted to change by substitution for Glu (Fig. 5E,F).

In contrast, for ttUMPK, the ADP molecule in the ATP‐binding site is predicted to change its position upon the substitution of Ser11 for Glu (Fig. 5C). The side chain of Ser11 interacts with the phosphate moiety of ADP in the native structure. In the S236E structure, however, the phosphate moiety might be moved to avoid steric clash with the side chain of Glu11. A similar situation is observed for ttIDH. Citric acid, a substrate analog of this enzyme, interacts with the side chain of Ser98 (Fig. 5D), but its position might be moved to avoid steric clash with the side chain of Glu98.

We further calculated the relative SASA of the mutated site and the free energy difference between the WT and mutant protein (ΔΔG) (Table 3). SASA analysis suggests that most of the phosphorylation sites are not exposed to the solvent and that substitution for Glu does not dramatically increase the SASA value. Mutations that introduce polar residues into the hydrophobic core can cause a significant loss of stability by >5 kcal·mol^−1^, often resulting in inactivation [29, 30]. However, the predicted ΔΔG values for the proteins examined in this study range from −0.5 to +0.5 kcal·mol^−1^ (Table 3). These results suggest that the substitution for Glu has little effect on their structural stability.

**Table 3:** Prediction of the effect of mutations.

Enzyme	Position	Wild‐type/Mutant	SASA (%)^a^	ΔΔG (kcal/mol)^b^
ttAMPK	34	Ser	9	
		Glu	19	0.15
		Ala	7	0.04
ttCMPK	11	Ser	9	
		Glu	14	−0.03
		Ala	7	0.05
ttUMPK	11	Ser	23	
		Glu	25	−0.15
		Ala	23	0.11
ttIDH	98	Ser	21	
		Glu	33	−0.41
		Ala	23	−0.37
ttMDH	236	Ser	55	
		Glu	71	−0.49
		Ala	62	−0.42
ttPPase	140	Tyr	11	
		Glu	9	0.2
		Phe	9	0.3

^a^ SASA values represent the relative per‐residue solvent accessible surface area between 0 (fully buried) and 100 (fully exposed). These are average values calculated for model structures predicted by AlphaFold3

^b^ ΔΔG values were calculated by STRUM. A ΔΔG below zero means that the mutation causes destabilization.

## Discussion

The activities of all phosphomimetic mutants analyzed in this study were markedly reduced. In NMPKs, the phosphomimetic mutations caused a large decrease in *k*_cat_. AMP kinase and CMP kinase belong to a different family from UMP kinase; they show no similarity in amino acid sequence or three‐dimensional structure. Nevertheless, the activities of both families of NMPKs were greatly reduced in the phosphomimetic forms. This suggests that the activity of all NMPKs belonging to different families can be reduced by phosphorylation near the active site. Structural prediction and energy calculation suggest no global structural changes caused by the phosphomimetic mutations. In fact, the *K*_m_ value was not altered by the mutation to Glu in ttCMPK. Therefore, the large decrease in *k*_cat_ is thought to be mainly due to the inhibition of the phosphoryl transfer reaction from ATP to nucleoside monophosphate. When a phosphomimetic mutation (Glu) is introduced into a Ser residue, it becomes negatively charged, and thus it is expected that it will repel negatively charged nucleotides. The results showed that the Ser‐to‐Ala mutation did not have a marked effect on the binding affinity of nucleotides (Table 2). Therefore, the introduction of a new negative charge may have physically or electrostatically changed the microenvironment of the active site, preventing the correct orientation of the phosphate group of the nucleotide. In the S11E structure of ttUMPK, the ADP molecule was predicted to change its position (Fig. 5C). It should be noted, however, that in the case of ttUMPK, the Ser‐to‐Ala mutation resulted in a drastic decrease in activity. This indicates the functional importance of Ser11, but from this assay, it seems difficult to estimate the effects of introducing a negative charge into this site. From another point of view, the phosphorylation site Ser11 of ttCMPK is located in a domain that moves when the three‐dimensional structure is in a closed conformation [18]. Therefore, it is possible that the introduced Glu residue hinders the movement of the domain, preventing other residues from taking the necessary position for activity.

**Fig. 4:**
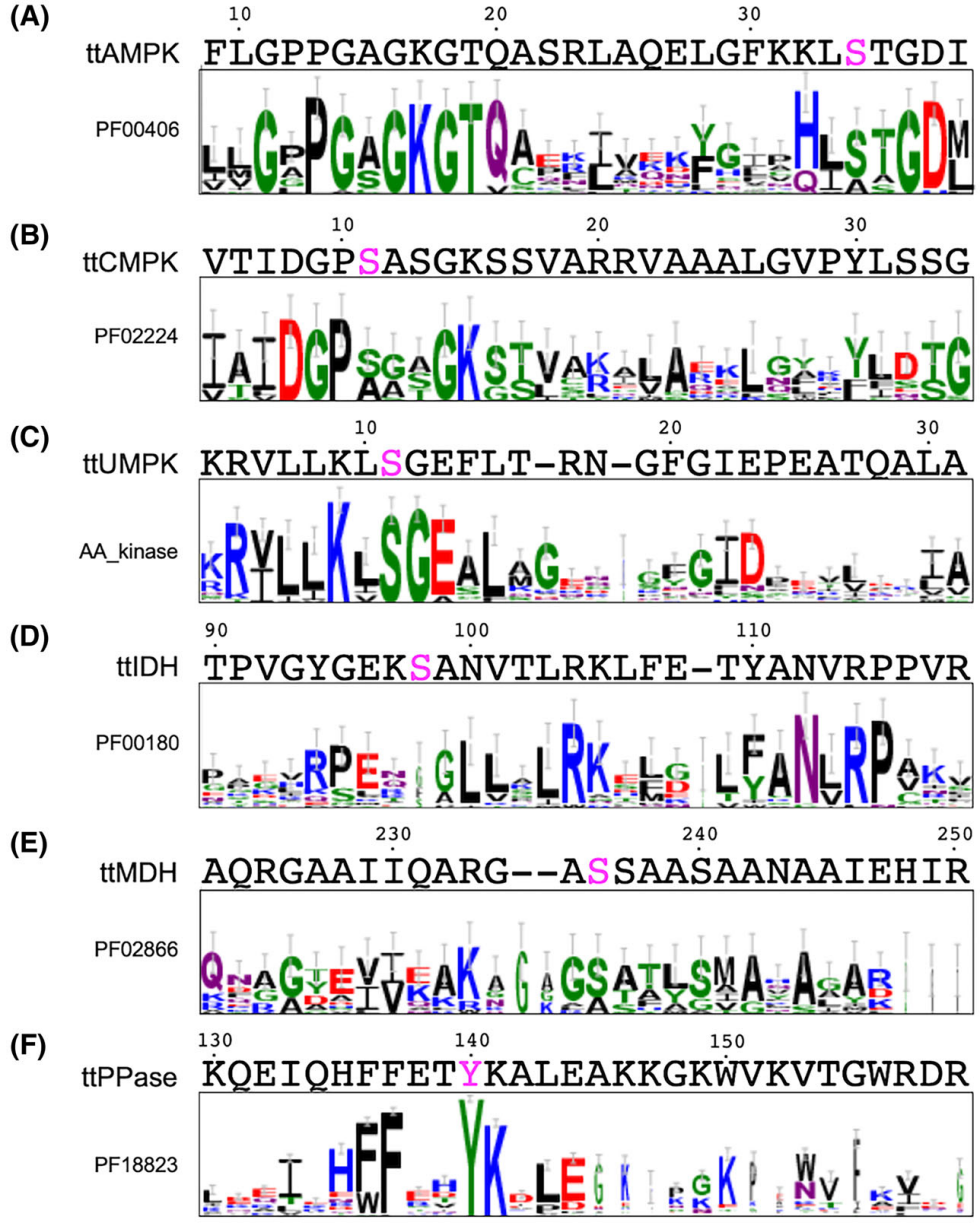
Sequence conservation at the phosphosites identified in *T. thermophilus* proteome. In each panel, the upper part shows the amino acid sequences of *T. thermophilus* enzymes, and the lower box shows the sequence logo of the respective protein (domain) families. The proteins are as follows: (A) AMP kinase, (B) CMP kinase, (C) UMP kinase, (D) isocitrate dehydrogenase, (E) malate dehydrogenase, and (F) inorganic pyrophosphatase. The phosphosites of the respective *Thermus* proteins are colored pink.

This Ser residue corresponding to the phosphosite of ttAMPK is relatively well conserved among AMP kinases (Fig. 4A). In addition, the phosphorylation of the Ser identified is relatively well conserved between bacterial and eukaryotic homologs. Phosphoproteome analysis has revealed phosphorylation of AMP kinase homologs of *E. coli* (Ser31) [2], *Mycobacterium bovis* (Ser30) [31], *Mycobacterium tuberculosis* Ser30 [32], *Staphylococcus aureus* Ser30 [33] and *Rhizobium meliloti* Ser30 [34]. Phosphorylation of the Ser residue corresponding to Ser34 of ttAMPK was also identified in human AK1 (Ser33) and AK3 (Ser37) based on the phosphorylation database PhosphoSitePlus [35]. CMP kinases of *E. coli* [36] and *Acinetobacter baumannii* [37] are phosphorylated at Ser14 and Ser12, respectively, which corresponds to Ser11 of ttCMPK. This Ser residue is fairly conserved among homologs (Fig. 4B). It should be mentioned that phosphorylation of the Ser residue (Ser33) near the ligand‐binding site in human CMPK1 (UMP‐CMP kinase) was also identified, although it does not correspond to the phosphosite of ttCMPK. In bacterial UMP kinases, the amino acid sequences that include the phosphosite (Ser11) of ttUMPK are highly conserved (Fig. 4C). The Ser residue corresponding to Ser11 of ttUMPK is phosphorylated in UMP kinases of *Bacillus subtilis* (Ser14) [38] and *Synechocystis* sp. PCC 6803 (Ser31) [39]. These results suggest that there may be a universal mechanism for regulating activity by phosphorylation of amino acid residues near the ligand‐binding site.

**Fig. 5:**
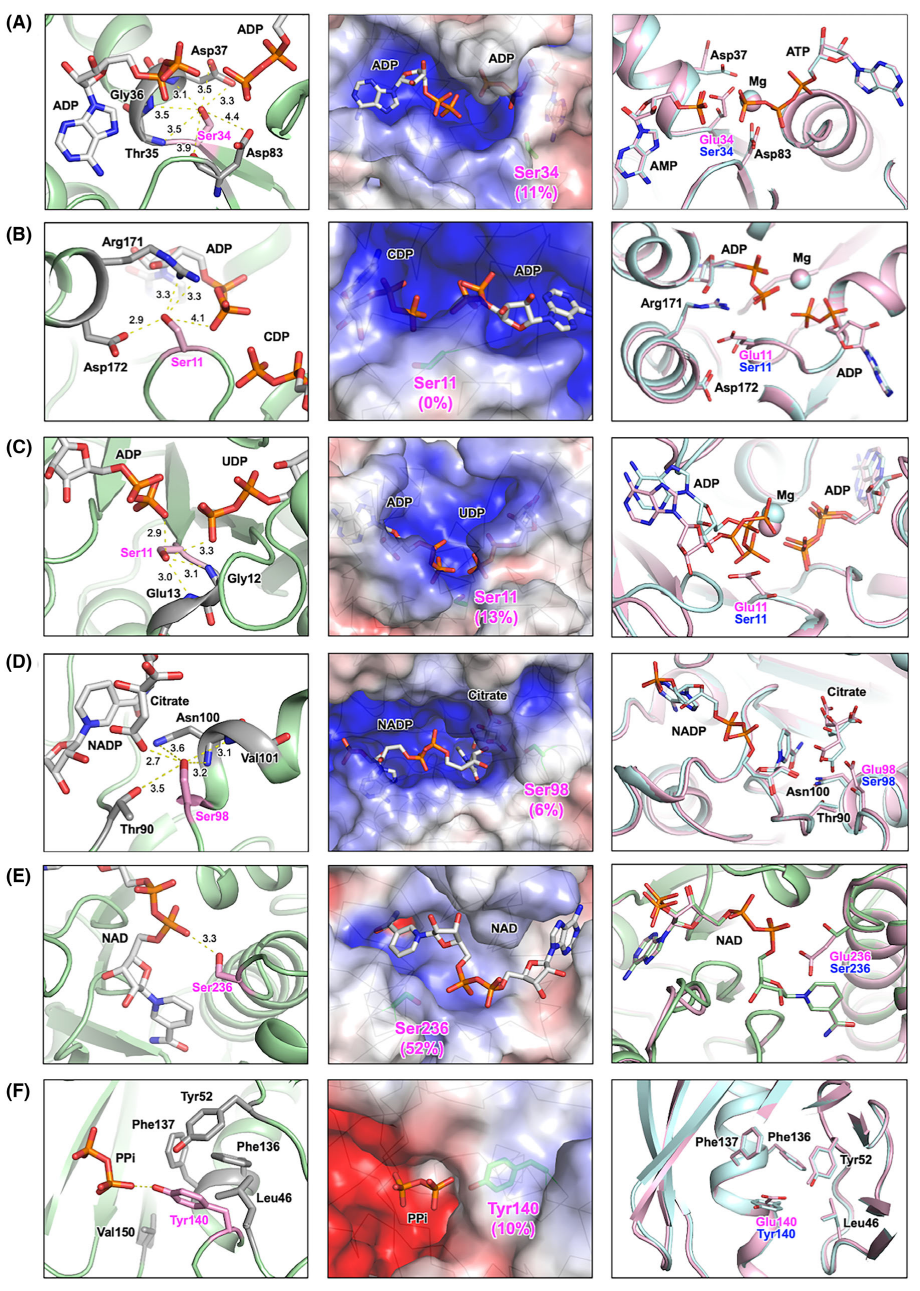
Structural characterization of the phosphosites. (A) ttAMPK, (B) ttCMPK, (C) ttUMPK, (D) ttIDH, (E) ttMDH, and (F) ttPPase. Left rows show possible interactions of the phosphosite with ligands and other residues. Middle rows show the local electrostatic potential map around the phosphosite. The percentage in parentheses is the relative per‐residue SASA in the structure shown. Right rows show superimposed model structures of the phosphomimetic mutant and WT proteins predicted by AlphaFold3.

The catalytic activities of ttIDH, ttMDH, and ttPPase were also affected by phosphomimetic mutation. In ttIDH, Ser98 interacts with citric acid and several other residues (Fig. 5D), suggesting its importance for the activity. This might be supported by the observation that the activity was reduced for both S98E and S98A mutant proteins. The S98E mutant protein showed much lower activity than S98A. In the predicted S98E structure, the position of citric acid was shifted, which might reduce the binding affinity to isocitrate, although most notable is the decrease in the *k*_cat_ value. In the case of ttMDH, even local structural change was not expected around residue 236 by the mutation (Fig. 5E), but both *K*_m_ and *k*_cat_ values decreased because of the mutation. For ttPPase, there was also a marked decrease in the *k*_cat_ value. These observed decreases in turnover number and substrate binding are thought to occur for the same reason as with NMPKs.

**Fig. 6:**
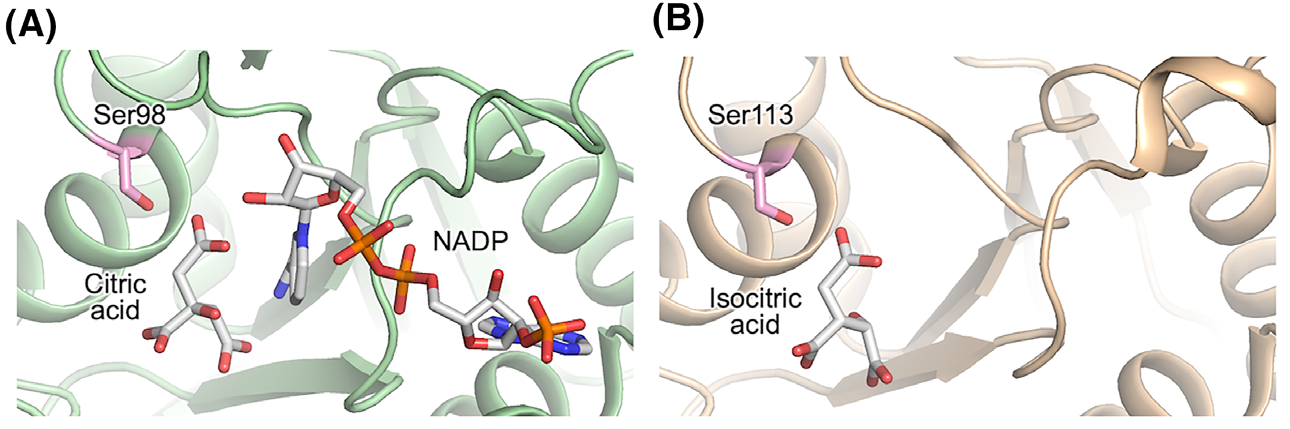
Phosphosites mapped on isocitrate dehydrogenase. (A) ttIDH (PDB ID, 2D1C). (B) *E. coli* isocitrate dehydrogenase (PDB ID, 1PB1). The side chain of the phosphosite Ser (colored in pink) and the bound ligands are shown as a stick model.

Malate dehydrogenase of *M. tuberculosis* is phosphorylated at Ser238 [40]. Also, in human and mouse cytoplasmic malate dehydrogenase 1 (Mdh1), Ser241 is phosphorylated [35]. These Ser residues correspond to Ser236 of ttMDH. The Ser residue at this position is well conserved among the homologs (Fig. 4E). Also, the Tyr residues corresponding to Tyr140 of ttPPase are well conserved. Inorganic pyrophosphatases of *E*. *coli* (Tyr142) [2], *M. tuberculosis* (Tyr126) [40] and *M. bovis* (Tyr126) [31] are phosphorylated at this site. Human inorganic pyrophosphatase PPA1 is phosphorylated at multiple sites, among which Tyr193 corresponds to Tyr140 of ttPPase [35]. As for isocitrate dehydrogenase, *B*. *subtilis* and *Pseudomonas aeruginosa* homologs are phosphorylated at Ser104 [41] and Ser115 [42], respectively, which correspond to Ser98 of ttIDH. This Ser residue is not well conserved among species (Fig. 4D). However, interestingly, the site (Ser113) in *E. coli* isocitrate dehydrogenase that corresponds to Ser98 in ttIDH is known to be phosphorylated (Fig. 6), and the mechanism of phosphorylation at this site and the fact that phosphorylation reduces activity have been elucidated [43]. This is a well‐known precedent for the regulation of an enzyme by phosphorylation at the active site [44]. In the *E. coli* enzyme, phosphorylation at Ser113 is catalyzed by the bifunctional isocitrate dehydrogenase kinase/phosphatase AceK [45]. Inactivation by phosphorylation of Ser113 and phosphomimetic mutation were reported to inhibit the binding of isocitrate [46, 47]. Our study also showed decreased affinity of isocitrate aligning with the phosphomimetic mutation, although the decrease in the catalysis was much more striking. It should be noted, however, that AceK has no homolog in *T. thermophilus*.

Protein phosphorylation has been considered to regulate protein activity through protein–protein interactions, protein localization, degradation, or allosteric regulation [48]. Most mechanisms of enzyme regulation by phosphorylation known to date involve conformational changes that affect activity at sites distant from the active site [49]. In contrast, our hypothesis of a regulatory mechanism via phosphorylation near the active site differs from known regulatory mechanisms in that phosphorylation can directly affect catalysis and possibly substrate binding. Compared with numerous examples, including glycogen phosphorylase and protein kinase, few examples are known of enzymes with regulating mechanisms involving phosphorylation of the active site. As mentioned above, the isocitrate dehydrogenase of *E. coli* provides one example in which phosphorylation inhibits catalytic activity by a direct effect within the substrate‐binding site [44]. The *E. coli* HipA phosphorylates a glutamyl‐tRNA synthetase GltX at its ATP‐binding site to inactivate it [50]. The *M*. *tuberculosis* β‐ketoacyl‐acyl carrier protein reductase MabA is phosphorylated at Thr191 and its phosphomimetic mutation markedly decreases the activity via reduced binding of NADPH [51]. Phosphorylation of the α subunit of the branched chain α‐ketoacid dehydrogenase complex at site 1 (Ser293) by a specific protein kinase inactivates the complex by the introduction of a negative charge [52]. The *M. tuberculosis* thymidylyltransferase RmlA is negatively regulated by phosphorylation at Thr residues located close to the catalytic triad [53]. Schastnaya et al. suggested that 44 of the 52 phosphosites play a regulatory role in *E. coli* metabolism and that the phosphorylation modulates the activity of several enzymes [54, 55].

We have not yet identified the *T. thermophilus* protein kinases responsible for phosphorylation of the sites examined in this study. As mentioned previously, these phosphosite residues are buried (Fig. 1) and not readily accessible to protein kinases [9, 10]. Structural rearrangements may occur to make such residues accessible for the kinase. Alternatively, kinase‐independent, nonenzymatic phosphorylation may occur in a similar way to nonenzymatic acetylation of lysine [56]. In addition, our preliminary investigations have shown that a part of the phosphorylation sites is located near ligand‐binding sites in other organisms. These points require further investigation, which is currently underway.

## Conflict of interest

The authors declare no conflict of interest.

## Author contributions

RM conceived and supervised the study. AN and RM designed the experiments. AN and HO performed the experiments. HO and YK provided MS tools and reagents. AN, HO, and RM analyzed the data. AN, HO, YK, and RM wrote the manuscript.

## Supporting information


**Fig. S1.** The pLDDT and PAE scores of the model structures of the phosphomimetic mutant proteins.

## Data Availability

The supporting data for the finding of this study are available in the supplementary material of this article.
