# Supplementary material for: Phosphomimetic mutations near active sites of proteins in Thermus thermophilus suggest a widespread regulatory mechanism
Source: FEBS Open Bio. 2025 Sep 15;15(12):1987–2000. doi: 10.1002/2211-5463.70103 (PMC12667214; doi:10.1002/2211-5463.70103)
Supplement: Supplementary file 1 — Fig. S1. The pLDDT and PAE scores of the model structures of the phosphomimetic mutant proteins. [file FEB4-15-1987-s001.docx]

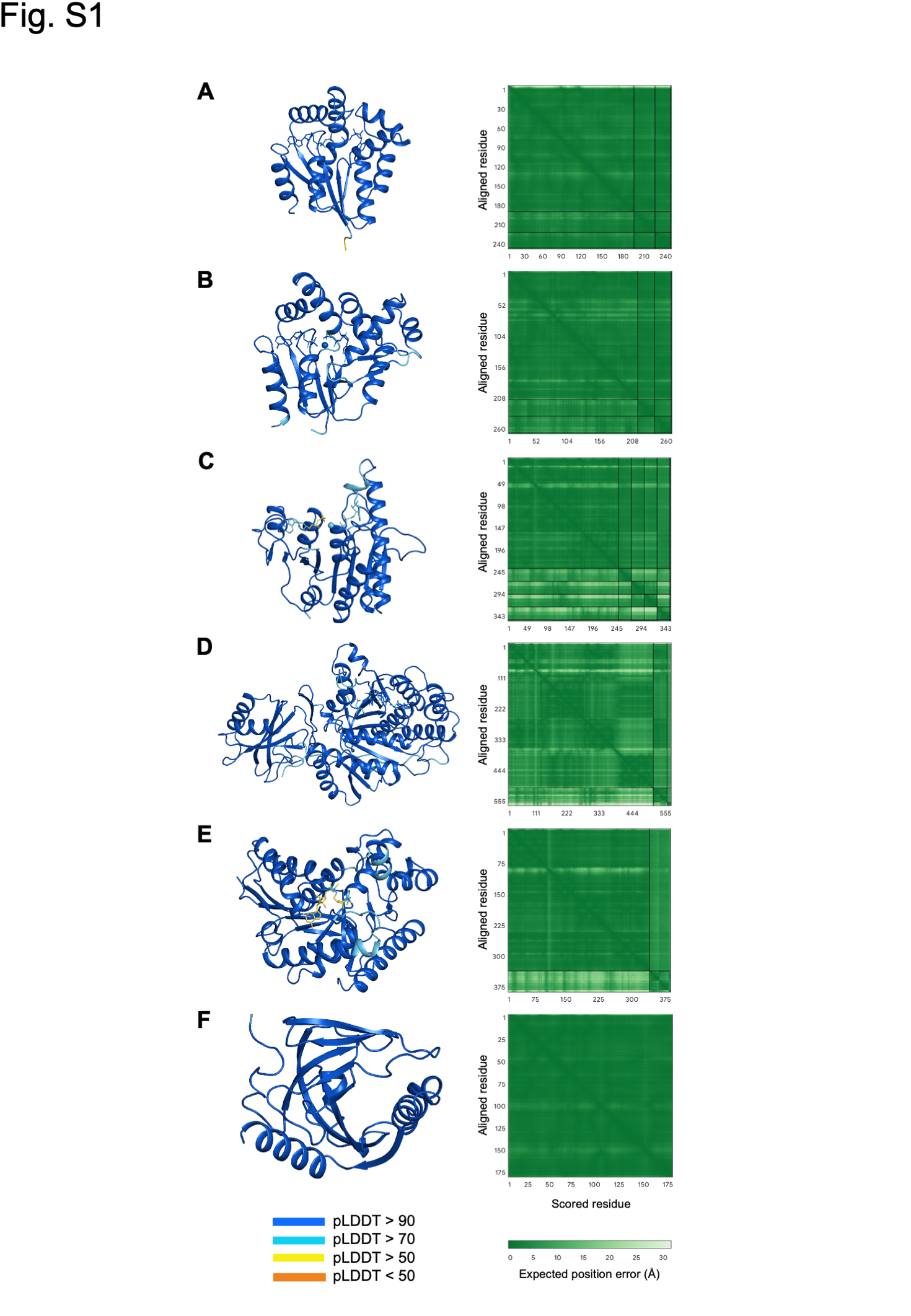


**Supporting Information Fig. S1. The pLDDT and PAE scores of the model structures of the phosphomimetic mutant proteins.**
